# Three novel piperidones exhibit tumor-selective cytotoxicity on leukemia cells via protein degradation and stress-mediated mechanisms

**DOI:** 10.1007/s43440-021-00322-3

**Published:** 2021-08-26

**Authors:** Lisett Contreras, Stephanie Medina, Austre Y. Schiaffino Bustamante, Edgar A. Borrego, Carlos A. Valenzuela, Umashankar Das, Subhas S. Karki, Jonathan R. Dimmock, Renato J. Aguilera

**Affiliations:** 1 Department of Biological Sciences and Border Biomedical Research Center, The University of Texas at El Paso, 500 West University Avenue, El Paso, TX 79968-0519 USA; 2 Drug Discovery and Development Research Group, College of Pharmacy and Nutrition, University of Saskatchewan, Saskatoon, S7N 5E5 Canada; 3 Department of Pharmaceutical Chemistry, Dr. Prabhakar B. Kore Basic Science Research Center, Off-Campus, KLE College of Pharmacy, (A Constituent Unit of KAHER-Belagavi), Bengaluru, Karnataka 560010 India

**Keywords:** Leukemia, Anticancer, Apoptosis, Proteasome inhibitor, Piperidone, Cancer

## Abstract

**Background:**

Cancer is an ongoing worldwide health problem. Although chemotherapy remains the mainstay therapy for cancer, it is not always effective and has detrimental side effects. Here, we present piperidone compounds P3, P4, and P5 that selectively target cancer cells via protein- and stress-mediated mechanisms.

**Methods:**

We assessed typical apoptotic markers including phosphatidylserine externalization, caspase-3 activation, and DNA fragmentation through flow cytometry. Then, specific markers of the intrinsic pathway of apoptosis including the depolarization of the mitochondria and the generation of reactive oxygen species (ROS) were investigated. Finally, we utilized western blot techniques, RT-qPCR, and observed the cell cycle profile after compound treatment to evaluate the possible behavior of these compounds as proteasome inhibitors. For statistical analyses, we employed the one-way ANOVA followed by Bonferroni post hoc test.

**Results:**

P3, P4, and P5 induce cytotoxic effects towards tumorigenic cells, as opposed to non-cancerous cells, at the low micromolar range. Compound treatment leads to the activation of the intrinsic pathway of apoptosis. The accumulation of poly-ubiquitinated proteins and the pro-apoptotic protein Noxa, both typically observed after proteasome inhibition, occurs after P3, P4, and P5 treatment. The stress-related genes *PMAIP1*, *ATF3*, *CHAC1*, *MYC*, and *HMOX-1* were differentially regulated to contribute to the cytotoxic activity of P3–P5. Finally, compound P5 causes cell cycle arrest at the G_2_/M phase.

**Conclusion:**

Taken together, compounds P3, P4, and P5 exhibit strong potential as anticancer drug candidates as shown by strong cytotoxic potential, activation of the intrinsic pathway of apoptosis, and show typical proteasome inhibitor characteristics.

**Supplementary Information:**

The online version contains supplementary material available at 10.1007/s43440-021-00322-3.

## Introduction

Cancer is the second leading cause of death worldwide and in the United States (U.S.) [1, 2]. In the U.S., disparities in cancer incidence are influenced by both socioeconomic status and race/ethnicity [3]. Moreover, several behavioral risk factors are associated with developing cancer, including smoking, diet, obesity, physical inactivity, and the absence of preventative care [3]. Therapies for the disease are rapidly evolving. In recent years, several treatments, including monoclonal antibody and Chimeric Antigen Receptor (CAR) T-cell therapy, have been explored with benefits that can resolve many facets of the disease [4, 5]. Nevertheless, chemotherapy remains the mainstay therapy for cancer [6]. It is typical for cancer cells to become resistant to current chemotherapeutic agents [6, 7]. Chemotherapy can also result in off-target toxicity of healthy cells [7]. Therefore, it is necessary to investigate new therapies to target cancer cells specifically.

Piperidones have been extensively studied in our laboratory as tumor-selective cytotoxic agents [8–11]. Previously, we reported two piperidone compounds, P1 and P2, which efficiently killed tumorigenic cells by apoptosis [8]. We detected several genes essential to these compounds' cytotoxic activity, and these were *PMAIP1*, *ATF3*, *CHAC1*, *MYC*, and *HMOX-1* [8]. These genes play a role in generating the cytotoxic effects induced by P1 and P2. Protein analysis revealed the accumulation of poly-ubiquitinated proteins, characteristic of proteasome inhibition [12]. In addition, the induction of the BH3-only pro-apoptotic protein Noxa (the product of *PMAIP1)*, which is known to accumulate during proteasome inhibition, was detected after compound exposure [8, 13]. We concluded that these compounds induce apoptosis through proteotoxic stress that is developed by the accumulation of misfolded/unfolded proteins characteristic of proteasome inhibition [8]. Proteasome inhibitors are therapeutic agents that can selectively target cancer cells [14]. This type of targeted therapy may alleviate the issues currently observed with chemotherapy [5].

**Fig. 1:**
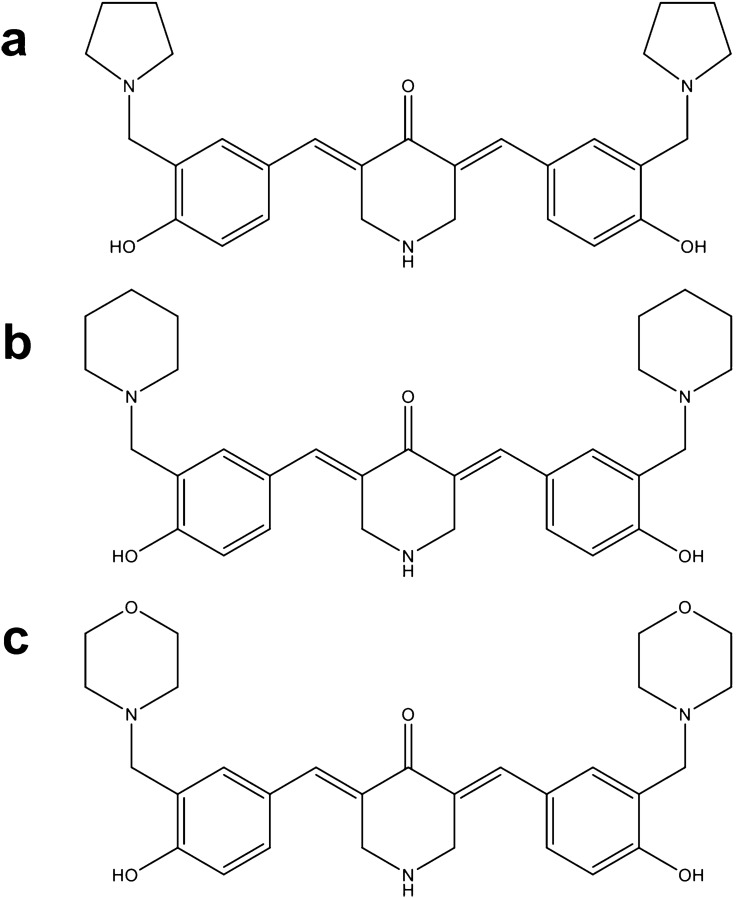
Structures of **a** P3, **b** P4, and **c** P5

Here, we present the characterization of three related piperidones (P3, P4, and P5; Fig. 1). In prior studies, P3, P4, and P5 showed potential as anticancer agents since they reduced the proliferation of human and murine leukemia cells at low micromolar concentrations [15]. Furthermore, tumor-selective cytotoxicity was observed towards oral cell carcinoma and leukemia cancer cells as opposed to non-cancerous cells (gingival fibroblasts, pulp cells, and periodontal ligament fibroblasts) [15]. In the current study, we investigate P3, P4, and P5's cytotoxic activity towards more cancer (lymphoma, leukemia, breast cancer, and colon cancer) and non-cancerous cell lines (fibroblasts and breast epithelial cells). Structural similarities can indicate a similar mode of action, as is the case with several compounds containing ɑ, β unsaturated keto moieties which behave as inhibitors of proteasome-associated deubiquitinases [16]. Therefore, we investigated the potential of these compounds to behave as proteasome inhibitors as we did with P1 and P2.

## Materials and methods

### Compound synthesis

Compounds were obtained from our collaborator’s library at the University of Saskatchewan. The compounds were prepared by a literature procedure [15]. In brief, a Mannich reaction was undertaken between 4-hydroxybenzaldehyde, formaldehyde, and the appropriate amine hydrochloride to afford the desired 3,4-disubstituted aryl aldehyde, which was condensed with 4-piperidone to give the desired products.

### Cell culture

All cell lines were obtained commercially from ATCC (American Type Culture Collection). Several cancer and non-cancerous cell lines were used for this analysis. Hematological cancer cell lines included CCRF-CEM (Acute Lymphoblastic Leukemia; CCL-119), HL-60 (Acute Myelocytic Leukemia; CCL-240), Jurkat (Acute Lymphocytic Leukemia; TIB-152), K562 (Chronic Myelogenous Leukemia; CCL-243), KCL22 (Chronic Myelogenous Leukemia; CRL-3350), and Ramos (Burkitt’s Lymphoma; CRL-1596), which were cultured under the same conditions with RPMI-1640 medium. Solid tumor cancer cell lines included MDA-MB-231 (Adenocarcinoma; HTB-26) cultured in DMEM medium, HT-29 (Colorectal Adenocarcinoma; HTB-38) cultured in McCoy’s 5A medium, and COLO 205 (Colorectal Adenocarcinoma; CCL-222) cultured in RPMI-1640 medium. Non-cancerous cell lines included Hs27 (No disease, foreskin; CRL-1634) cultured in DMEM medium and MCF-10A (Fibrocystic Disease; CRL-10317) cultured in DMEM/F12 medium supplemented with 20 ng/ml epidermal growth factor, 0.5 µg/ml hydrocortisone, and 10 µg/ml insulin. Additionally, all media used for cell culture was supplemented with 10% heat-inactivated fetal bovine serum (FBS), and a mixture of 25 µg/ml amphotericin B, 1,000 U/ml penicillin, and 1,000 µg/ml streptomycin. The HL-60 cell line was supplemented with 20% FBS and the same antibiotic mixture described. All cell lines were grown in an incubator supplemented with 5% CO_2_ at 37 °C.

### Differential nuclear staining (DNS) assay

Cytotoxic activity was evaluated through the DNS assay [17, 18]. This assay involves labeling live and dead cells with two nucleic acid intercalators, Hoechst 33342 and propidium iodide (PI). Hoechst 33342 stains all cells (healthy and dead), but PI stains only dead or dying cells with a compromised cell membrane [17, 18]. For the experiment, each cell line was plated at a density of 10,000 cells per well (96-well plate) in 100 µl media and placed in the incubator overnight under cell culture conditions. Cells were then treated with varying concentrations of the compound of interest for 48 h. Two hours before incubation elapsed, 10 µl of a dye mixture consisting of phosphate-buffered saline (PBS), Hoechst 33342 (1 μg/ml final; Invitrogen, H1399), and PI (1 μg/ml final; Invitrogen, P1304MP) was added to each well. The IN Cell 2000 analyzer, a high-content analyzer (HCA), was used to image the plates (GE Healthcare). For quantitative analysis, images were segmented through the IN Cell Analyzer Workstation 3.2 software (GE Healthcare). Cytotoxic concentration 50% (CC_50_) values were calculated using a linear interpolation method previously described [19]. CC_50_ is defined as the concentration of compound needed to kill 50% of the cell population [19]. Each plate had the same set of controls that included 1% v*/*v DMSO as vehicle control, 1 mM hydrogen peroxide (H_2_O_2_) as a positive control for death, and untreated cells as a negative control. Each compound concentration and each control were assessed in triplicate. The selective cytotoxicity index (SCI) is defined as a compound's ability to preferentially kill cancer cells as opposed to non-cancerous cells [20]. SCI was calculated using the following equation: CC_50_ of the non-cancer cell line/CC_50_ of the cancer cell line [20].

### Concentrations for cell death induction

For the experiments conducted, the concentrations used correspond to either CC_50_ or CC_50_ × 2 in the specified cell line. In HL-60, compound P3 has a CC_50_ of 1.7 µM and a CC_50_ × 2 of 3.4 µM, compound P4 has a CC_50_ of 2 µM and a CC_50_ × 2 of 4 µM, and compound P5 had a CC_50_ of 2 µM and a CC_50_ × 2 of 4 µM. For the cell cycle analysis, lower concentrations were used to observe the effects of the compound as the cell divides over a longer period (72 h). Therefore, we used a CC_10_ and a CC_30_ concentration. The concentrations used for P3 treatment were 0.34 µM (CC_10_) and 1.02 µM (CC_30_), for P4 treatment were 0.4 µM (CC_10_) and 1.2 µM (CC_30_), and for P5 treatment were 0.4 µM (CC_10_) and 1.2 µM (CC_30_).

### Annexin V-FITC assay

The annexin V-FITC assay was used to evaluate the induction of apoptosis (Beckman Coulter; IM3546) [21]. HL-60 cells were seeded at a density of 100,000 cells in 1 ml complete medium. Cells were treated with the three compounds of interest for 24 h. Concentrations used for P3 were 1.7 µM and 3.4 µM, for P4 were 2 µM and 4 µM, and for P5 were 2 µM and 4 µM. Controls used were a vehicle control (0.1% v*/*v DMSO), an apoptosis-inducing positive control (1 mM H_2_O_2_), and an untreated cell negative control. Following treatment, cells were collected and stained with both PI and annexin V-FITC according to the manufacturer’s instructions (Beckman Coulter; IM3546). Samples were immediately read by flow cytometry (Gallios; Beckman Coulter). Data analysis was accomplished with Kaluza software (Kaluza Analysis Software; Beckman Coulter).

### Caspase-3 assay

The activation of caspase-3 was detected using the fluorogenic NucView 488 caspase-3 substrate, which identifies active caspase-3 within live cells (Biotium; 30029). The percentage of cells emitting a green fluorescent signal, observed through flow cytometry, were counted as cells with active caspase-3. HL-60 cells were plated in a 24-well plate at a density of 100,000 cells/1 ml/well. Cells were treated for 8 h with P3 at 1.7 µM and 3.4 µM, P4 at 2 µM and 4 µM, and P5 at 2 µM and 4 µM. The same controls as mentioned previously were used (0.1% v/v DMSO, 1 mM H_2_O_2_, and untreated cells). After incubation, samples were collected and stained according to the manufacturer’s protocol. Samples were then analyzed via flow cytometry (Gallios & Kaluza Analysis Software; Beckman Coulter).

### Mitochondrial membrane potential (ΔΨm) polychromatic assay

The cationic JC-1 (5′,6,6′-tetrachloro-1,1′,3,3′-tetraethylbenzimidazolylcarbocyanine iodide) dye can be used to monitor mitochondrial membrane potential (Invitrogen; M34152). A red signal (JC-1 aggregates) indicates an intact polarized mitochondrial membrane, while a green signal (JC-1 monomers) indicates a depolarized mitochondrial membrane. HL-60 cells were plated in a 24-well plate (100,000 cells/well) and treated with P3 (1.7 µM and 3.4 µM), P4 (2 µM and 4 µM), and P5 (2 µM and 4 µM) for 5 h. A vehicle, positive, and negative control (as previously described) were included as well. Following incubation, cells were collected and stained per the manufacturer’s instructions with a final concentration of 2 µM JC-1 dye. Samples were then examined utilizing flow cytometry (Gallios; Beckman Coulter). Analysis of the subsequent data was completed using Kaluza software (Kaluza Analysis Software; Beckman Coulter).

### Reactive oxygen species (ROS) assay

Reactive oxygen species (ROS) generation can be quantified using the 6-carboxy-2',7'-dichlorodihydrofluorescein diacetate (carboxy-H2DCFDA) dye (Invitrogen; Molecular Probes, C400) [19, 22]. This non-fluorescent dye shifts to a green fluorescent form as oxidation, induced by ROS, occurs within the cell. Therefore, a green fluorescent signal, corresponding to the oxidized form of carboxy-H2DCFDA, indicates ROS generation. Cells (HL-60) were plated overnight at a density of 100,000 cells in 1 ml. The next morning, cells were treated with P3 at 1.7 µM and 3.4 µM, P4 at 2 µM and 4 µM, and P5 at 2 µM and 4 µM for 18 h. Samples were collected, centrifuged at 262*g* for 5 min, and re-suspended in 1 ml PBS to remove the complete medium. Then, cells were loaded with a final concentration of 10 µM carboxy-H2DCFDA dye for 45 min at 37 °C. Following incubation with dye, cells were centrifuged at 262*g* for 5 min and re-suspended in 500 µl PBS. Then, samples were processed using flow cytometry (Gallios; Beckman Coulter). Data analysis was completed with the Kaluza software (Kaluza Analysis Software; Beckman Coulter). Controls used were the same as mentioned before (0.1% v*/*v DMSO, 1 mM H_2_O_2_, and untreated cells) with an additional unstained (not loaded with dye) control to observe the normal population of cells.

### Cell cycle analysis with NIM-DAPI

The phases of the cell cycle can be visualized through flow cytometry by measuring the amount of DNA content of cells [23]. The DNA intercalating fluorophore 4, 6-Diamidino-2-phenylindole (DAPI) can be used to stain DNA. Cells can be permeabilized and stained using a nuclear isolation medium (NIM)-DAPI solution (Beckman Coulter) to quantify DNA content [17, 19]. HL-60 cells (100,000 cells/ml) were treated with 0.34 µM and 1.02 µM of P3, 0.4 µM and 1.2 µM of P4, and 0.4 µM and 1.2 µM of P5 for 72 h. Controls included were a vehicle (0.1% v*/*v DMSO), positive (1 mM H_2_O_2_), and negative (untreated cells) control. Subsequently, cells were collected in flow cytometry tubes and centrifuged at 262*g* for 5 min. Then, the supernatant was removed, and the cell pellet was re-suspended in a mixture of 100 µl of PBS and 200 µl of NIM-DAPI. Samples were immediately read and analyzed through flow cytometry (Gallios & Kaluza Analysis Software; Beckman Coulter). Approximately 10,000 events were acquired per sample. Data are represented as plots that display peaks (Fig. 6e) which represent fragments of DNA (Sub-G_0/1_ phase), cells before they replicate DNA (G_0/1_ phase), active replication of DNA (S phase), and post-replicative stage while entering mitosis (G_2_/M phase) [23].

### Western blot

HL-60 cells (3,000,000) were treated with P3 (3.4 µM), P4 (4 µM), P5 (4 µM), and a vehicle control (0.3% v*/*v PEG-400) for 8 h. Then, 70 µl of 2 × Laemmli buffer (120 mM Tris–HCl, 0.1% β-mercaptoethanol, 4% SDS, 20% glycerol, and 0.02% v*/*v bromophenol blue) were added to dry cell pellets and boiled for 10 min at 100 °C to extract protein. The NanoDrop 1000 system (Thermo Fischer) was used to quantify protein content. A concentration of 100 µg protein in a final volume of 25 µl was loaded per lane on a 10% SDS polyacrylamide gel. Proteins were separated for 1 h at 100 V, then transferred by wet transfer to a polyvinylidene fluoride (PVDF) membrane for 1 h at 100 V. Membranes were blocked overnight at 4 °C in a 5% milk/TBS-T (Tris-Buffered Saline-0.001% Tween) solution. Then, membranes were incubated with primary antibody for 1 h at room temperature. Primary antibodies used were mouse monoclonal anti-ubiquitin (1:1,000 dilution; Santa Cruz Biotech, sc-8017) and mouse monoclonal anti-Noxa (1:1,000; Thermo Fischer, MA1–41000) diluted in a 5% Bovine Serum Albumin (BSA)/TBS-T solution and a mouse monoclonal anti-β actin conjugated to horseradish peroxidase (1:25,000 dilution; Sigma-Aldrich A3854) diluted in a 5% milk/TBS-T solution. The secondary antibody used was polyclonal goat anti-mouse conjugated to horseradish peroxidase (1:10,000 dilution; Thermo Scientific) diluted in TBS-T. Images were obtained using the Thermo Fischer iBright 1500 instrument in the Genomic Analysis Core Facility at the Border Biomedical Research Center (BBRC) at the University of Texas at El Paso (UTEP). The final figure shows blots that were cropped to focus on the area of interest. Original, unedited blots can be found in Supplementary File 2. Densitometry analysis was accomplished using the Image Studio Lite (LI-COR) software.

### Reverse transcriptase real-time polymerase chain reaction (RT-qPCR)

A total of 1,000,000 HL-60 cells (plated at a density of 200,000 cells per ml) were treated with P3 (3.4 µM), P4 (4 µM), P5 (4 µM), and a vehicle control (0.3 v*/*v PEG-400) for 6 h. After the incubation period elapsed, cell pellets were washed by centrifuging at 262*g* for 5 min and re-suspending in 1 ml of PBS. Dry cell pellets were used for RNA extraction. RNA was extracted immediately according to the manufacturer’s instructions of the RNeasy Mini Kit (Qiagen; 74104). A QIAshredder was used to lyse the cells, and the optional DNase I digestion was also accomplished. Three biological replicates were used for each treatment. RNA was stored at -80 ºC overnight, then the RNA was used to synthesize cDNA using the RT^2^ HT First Strand Kit (Qiagen; 330,411). The amount of RNA used per sample was 500 ng, which was quantified using a NanoDrop ND-1000 system (ThermoFisher Scientific). RNA was diluted using RNase-free water to obtain the desired concentration (500 ng) in 9 µl. As per the manufacturer’s protocol, we incubated 9 µl of the sample with 6 µl GE2 (gDNA elimination buffer) for 5 min at 37 °C, then we added 6 µl BC4 Reverse Transcriptase Mix and incubated for 15 min at 42 °C, 5 min at 95 °C and, finally, a 4 °C hold. Then, 91 µl of RNase-free water was added to each sample for a final concentration of 4.5 ng/µl. The generated cDNA was then stored at − 20 °C. An iCycler Thermal Cycler (Bio-Rad; 582BR) was used to carry out real-time polymerase chain reactions (qPCR). Each qPCR reaction (25 µl total volume) contained the following components: 12.5 µl RT^2^ SYBR Green (Qiagen; 330512), 3.5 µl forward (sense) primer (Bioneer), 3.5 µl reverse (antisense) primer (Bioneer), 1.5 µl nuclease-free water, and 4 µl cDNA template. For each reaction, samples were tested in triplicate (technical replicates) along with a negative control (nuclease-free water). Three independent experiments were conducted for each gene. For data analysis, gene expression levels were normalized to the housekeeping gene (*ACTB*) and quantified using the comparative C_t_ method (2^−∆∆Ct^). Fold change differences were calculated by comparing gene expression levels of compound treatment versus vehicle control. PCR cycles and details of primer sequences were obtained from a published methodology (see Supplementary Table 1) [8].

### In silico screening

The compounds were prepared using the Ligprep interface of the Schrodinger software [24] with an OPLS3 force field at a pH 7 ± 2 using Epik [25]. The other options were set to the default of the Schrodinger software. The preparation of proteins was done as previously described [26]. Protein’s crystal structures were obtained from the Protein Data Bank (PDB) (https://www.rcsb.org/). For UCHL5 PDB: 3RII, 4UEM, and 4UF5 were used, and for USP14 PDB: 2AYN, 2AYO, and 6IIN were used. Receptor grid generation was defined by using the Sitemap tool in the Schrodinger software [27], the best scoring sites were selected for the receptor grid generation tool in maestro 11.5. Molecular docking was performed using the Glide tool on maestro 11.5 [28] using the standard precision (SP) algorithm. Finally, molecular mechanics (MM-GBSA) of the docked compounds was performed using the Prime tool on maestro 11.5 [29].

### Statistical analysis

The data obtained were analyzed using Microsoft Excel® and an online calculator (https://astatsa.com/OneWay_Anova_with_TukeyHSD/). Data are presented as the mean value (*n* = 3, except where noted) for each experiment ± standard deviation (SD). Statistically significant differences between treatment and control groups were assessed by one-way analysis of variance (ANOVA), followed by Bonferroni post hoc test. For all analyses, a *p*-value ≤ 0.05 was considered significant.

## Results

### Tumor selective cytotoxicity is observed with treatment of P3, P4, and P5

The DNS assay was utilized to measure the cytotoxic ability of compounds P3, P4, and P5. The compounds of interest were tested at a 48-h time point in nine cancerous cell lines and two non-cancerous cell lines. After these assays, we calculated the cytotoxic concentration at which 50% of the cell population is dead (CC_50_) for each compound in each cell line. The average CC_50_ values of P3–P5 towards the nine tumorigenic cell lines listed in Table 1 are 2.26 µM, 1.91 µM, and 1.52 µM, respectively. The most sensitive cell line to P3 and P4 treatment was HL-60 and to P5 treatment was CCRF-CEM, both hematological cancer cell lines. An important feature of candidate cytotoxins is to exert a greater cytotoxic effect on neoplasms than on normal cells. Therefore, P3–P5 were screened against non-malignant Hs27 and MCF-10A cells where the results are presented in Table 1. We observed selective cytotoxicity (SCI above 1) towards cancerous cells as opposed to non-cancerous cells with the treatment of all three compounds (Table 1). The average CC_50_ values for P3–P5 towards Hs27 and MCF-10A are 4.99, 3.83, and 3.84 µM, respectively, indicating the normal cells' greater tolerance to P3 than to P4 and P5. Melphalan is an established anticancer drug whose efficacy towards the nine tumorigenic cell lines is presented in Table 2. Our compounds of interest revealed improved cytotoxicity when compared to the cytotoxicity of melphalan. P3, P4, and P5 are 7.88, 9.32, and 11.7 times, respectively, more potent than melphalan, which exhibited an average CC_50_ value of 17.8 µM.

**Table 1:** CC_50_ determination of compounds P3, P4, and P5 on various cancerous and non-cancerous cell lines

Cell line	Disease	P3			P4			P5		
		^a^CC_50_	SD	^b^SCI	CC_50_	SD	SCI	CC_50_	SD	SCI
CCRF-CEM	Acute lymphocytic leukemia (ALL)	0.87	0.02	9.47	0.75	0.02	5.48	0.65	0.06	8.42
COLO 205	Colorectal adenocarcinoma	4.66	0.38	1.77	2.79	0.29	1.48	0.80	0.14	6.87
HL-60	Acute promyelocytic Leukemia	0.59	0.05	13.92	0.64	0.07	6.51	0.74	0.01	7.47
Hs27	None	8.23	0.49	–	4.13	0.27	–	5.50	0.89	–
HT-29	Colorectal adenocarcinoma	0.89	0.09	9.21	0.79	0.06	5.26	1.93	0.08	2.85
Jurkat	Acute T-cell leukemia	0.96	0.03	8.57	0.97	0.02	4.26	0.91	0.02	6.05
K562	Chronic myelogenous leukemia (CML)	9.05	0.93	0.91	8.37	0.92	0.49	4.25	0.99	1.29
KCL22	Chronic myelogenous leukemia (CML)	0.87	0.10	9.44	0.93	0.14	4.47	1.57	0.22	3.49
MCF-10A	Fybrocystic disease	1.75	0.21	–	3.52	0.17	–	2.18	0.13	–
MDA-MB-231^c^	Adenocarcinoma	1.52	0.13	1.15	0.99	0.08	3.55	1.74	0.07	1.26
Ramos	Burkitt’s lymphoma	0.94	0.08	8.75	1.00	0.10	4.13	1.06	0.13	5.19

^a^CC_50_ values in µM

^b^Selective Cytotoxicity Index (SCI) was calculated using the following equation: CC_50_ of non-cancer cells divided by the CC_50_ of the cancer cell line. Values above 1 indicate selective cytotoxicity of the compound towards cancer cells

^c^SCI calculated using MCF-10A CC_50_ (breast epithelial). All others using Hs27

**Table 2:** Cytotoxic evaluation (CC_50_) of melphalan in various cancerous cell lines

Cell line	Disease	Melphalan	
		CC_50_ (µM)	SD
CCRF-CEM	Acute lymphocytic leukemia (ALL)	3.17	0.62
COLO 205	Colorectal adenocarcinoma	20.40	0.67
HL-60	Acute promyelocytic leukemia	1.57	0.27
HT-29	Colorectal adenocarcinoma	21.07	1.86
Jurkat	Acute T-cell leukemia	1.91	0.23
K562	Chronic myelogenous leukemia (CML)	19.63	0.55
KCL22	Chronic myelogenous leukemia (CML)	81.28	5.65
MDA-MB-231	Adenocarcinoma	9.23	1.68
Ramos	Burkitt’s lymphoma	2.05	0.39

### Compounds P3–P5 induce the cell death mechanism of apoptosis, specifically the intrinsic pathway of apoptosis

**Fig. 2:**
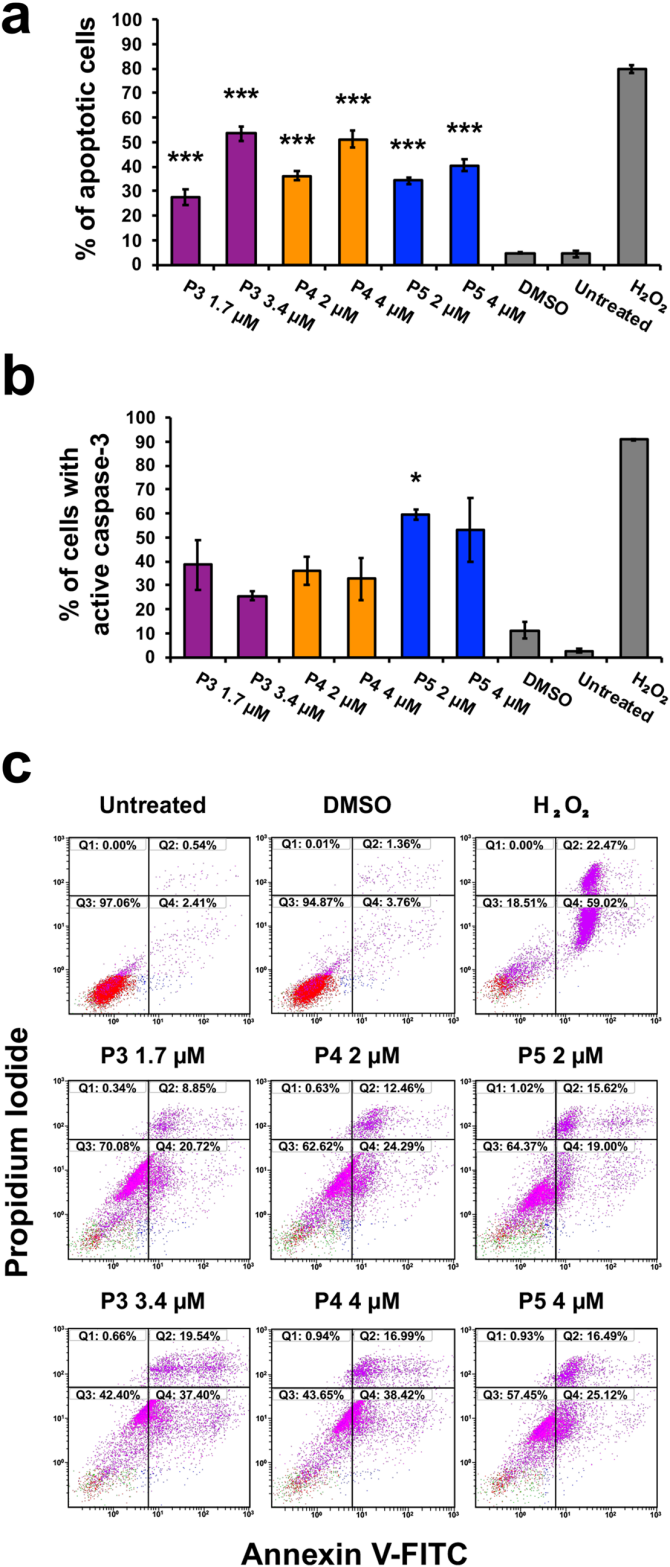
Treatment with P3, P4, and P5 causes the induction of apoptosis in HL-60 cells. **a** The annexin V-FITC assay was employed to detect phosphatidylserine externalization. The percentage of cells emitting a fluorescent signal from the annexin V-FITC complex bound to phosphatidylserine was considered apoptotic. HL-60 cells were treated with the indicated concentrations for 24 h. **b** A complex containing a caspase-3 substrate attached to a DNA dye was used to evaluate the activation of caspase-3. A green fluorescent signal is emitted when caspase-3 cleaves the DNA dye from the caspase-3 substrate. The percent of cells with active caspase-3, i.e., green fluorescence signal, was detected after 8-h treatment with compounds of interest. **c** Representative plots from flow cytometer analysis (approximately 10,000 events per sample) depicting Annexin V-FITC and propidium iodide (PI) staining. The following controls were included: a vehicle control (0.1% v*/*v DMSO), an apoptosis-inducing positive control (1 mM H_2_O_2_), and a negative control of untreated cells. The data represent the mean ± SD (*n* = 3, except for DMSO in (**b**) where *n* = 2). One-way ANOVA followed by Bonferroni post hoc test were used to assess significant differences between the treatment groups and the vehicle control (*p < 0.05, ***p < 0.0001)

To investigate the induction of apoptosis, we examined phosphatidylserine externalization and the activation of caspase-3. Phosphatidylserine is exposed as a cell goes through apoptosis [30]. Annexin V-FITC can bind to exposed phosphatidylserine to quantify the percentage of apoptotic cells [30]. Treatment with P3–P5 caused a significant increase of Annexin V-FITC-positive cells in comparison to the DMSO control, as assessed by one-way ANOVA (*F*_6,14_ = 129.10, *p* < 0.0001). Bonferroni post hoc analysis revealed significant differences between P3, P4, and P5 treatment versus the DMSO control (Fig. 2a). A significant increase of ~ 23%, ~ 32%, and ~ 30% Annexin V-FITC-positive cells, respectively, after P3, P4, and P5 treatment at the CC_50_ concentration was observed (Fig. 2a). Increasing the dose of compounds P3 and P4 induced a significant increase of ~ 49% and ~ 46% Annexin V-FITC-positive cells, respectively, in comparison to DMSO (Fig. 2a). Caspase-3 is an executioner caspase involved in the apoptosis cascade [30]. The percentage of cells with active caspase-3, indicated by a green fluorescent signal, can be determined using flow cytometry and a caspase-3/fluorogenic dye substrate. Analysis by one-way ANOVA revealed significant differences in caspase-3 activation between treatment and control groups (*F*_6,13_ = 3.60, *p* = 0.03). P3- and P4 treatment increased caspase-3 activation in ~ 27% and ~ 19% of cells, respectively, as compared to DMSO (Fig. 2b). However, the post hoc analysis revealed a significant increase only after P5 treatment with ~ 49% of cells showing active caspase-3 (Fig. 2b).

**Fig. 3:**
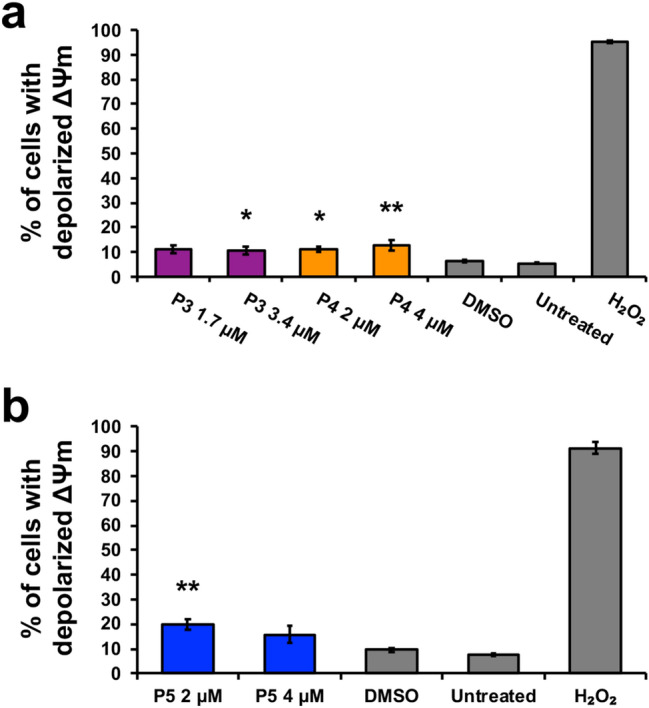
Compounds P3, P4, and P5 cause mitochondrial membrane potential depolarization that leads to the activation of the intrinsic pathway of apoptosis. The JC-1 dye was utilized to observe changes in mitochondrial membrane potential (ΔΨm). The monomer form of the JC-1 dye, indicating depolarized mitochondria, emits a green fluorescent signal that is visualized through flow cytometry. **a**, **b** Bars (mean ± SD, n = 3) represent the percentage of the HL-60 cell population with depolarized mitochondria after 5-h treatment with P3, P4, and P5. Controls included vehicle (0.1% v/v DMSO), an apoptosis-inducing positive control (1 mM H_2_O_2_), and untreated cells. Significant differences between compound treatment and the vehicle control were assessed by one-way ANOVA followed by Bonferroni post hoc analysis (*p < 0.05 and **p < 0.01)

**Fig. 4:**
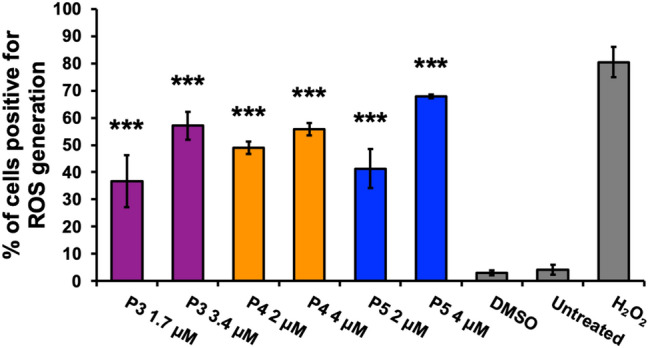
Generation of ROS is observed after treatment with compounds P3, P4, and P5 further verifying the activation of the intrinsic pathway of apoptosis. Reactive oxygen species (ROS) were detected via oxidation of the carboxy-H2DCFDA dye which emits a green fluorescent signal when ROS are generated. The percentage of cells (HL-60) positive for ROS accumulation after 18-h incubation with compounds P3, P4, and P5 is shown in the bar graph. An unstained control was used as a baseline and subtracted from each treatment value. Data are presented as a means ± SD (*n* = 3). A vehicle control (0.1% v*/*v DMSO), a positive control (1 mM H_2_O_2_), and a negative control of untreated cells were used for this experiment. One-way ANOVA test followed by Bonferroni post hoc analysis were employed to assess significant differences of the treatment groups compared to the vehicle control (***p < 0.0001)

The intrinsic and extrinsic pathways are the two main pathways of apoptosis [30]. The intrinsic pathway involves cellular stress, the generation of ROS, and changes in the mitochondrial membrane potential (ΔΨm) [31]. Utilizing the JC-1 dye, the mitochondrial membrane potential was monitored [31]. The monomer form of the JC-1 dye, emitting a green fluorescent signal, indicates depolarized mitochondrial membrane potential [31]. A significant difference in the percentage of cells with depolarized mitochondrial membrane potential between P3- and P4-treatment groups and DMSO (*F*_4,10_ = 7.06, *p* = 0.006), as well as between P5 treatment and DMSO (*F*_2,6_ = 11.53, *p* = 0.009) was observed. Bonferroni post hoc analysis revealed a significant increase in the cell population with a depolarized mitochondrial membrane of ~ 5% after P3 treatment at 1.7 µM, ~ 5% after P4 treatment at 2 µM, ~ 6% after P4 treatment at 4 µM, and ~ 10% after P5 treatment at 4 µM in comparison to DMSO (Fig. 3a, b). ROS generation was evaluated using the dye carboxy-H2DCFDA [22]. The oxidized form of carboxy-H2DCFDA, green fluorescent signal, indicates ROS production [22]. Significant differences, assessed by one-way ANOVA, between compound treatment groups and the DMSO control were observed in the percentage of green-positive or ROS-producing cells (*F*_6,14_ = 50.77, *p* < 0.0001). Post hoc analysis revealed a significant increase of ~ 34%, ~ 46%, and ~ 38% of cells positive for ROS production after P3, P4, and P5 treatment, respectively (Fig. 4). Additionally, treatment with twice the concentration of P3, P4, and P5 displayed significant differences of 54%, ~ 53%, and ~ 65% of cells positive for ROS production, respectively, as compared to DMSO (Fig. 4). Finally, dose dependence was observed after P3 and P5 treatment (Fig. 4).

### Cellular modulations characteristic of proteasome inhibition, including accumulation of poly-ubiquitinated proteins and the protein Noxa, are observed after P3–P5 treatment in leukemia cells

**Fig. 5:**
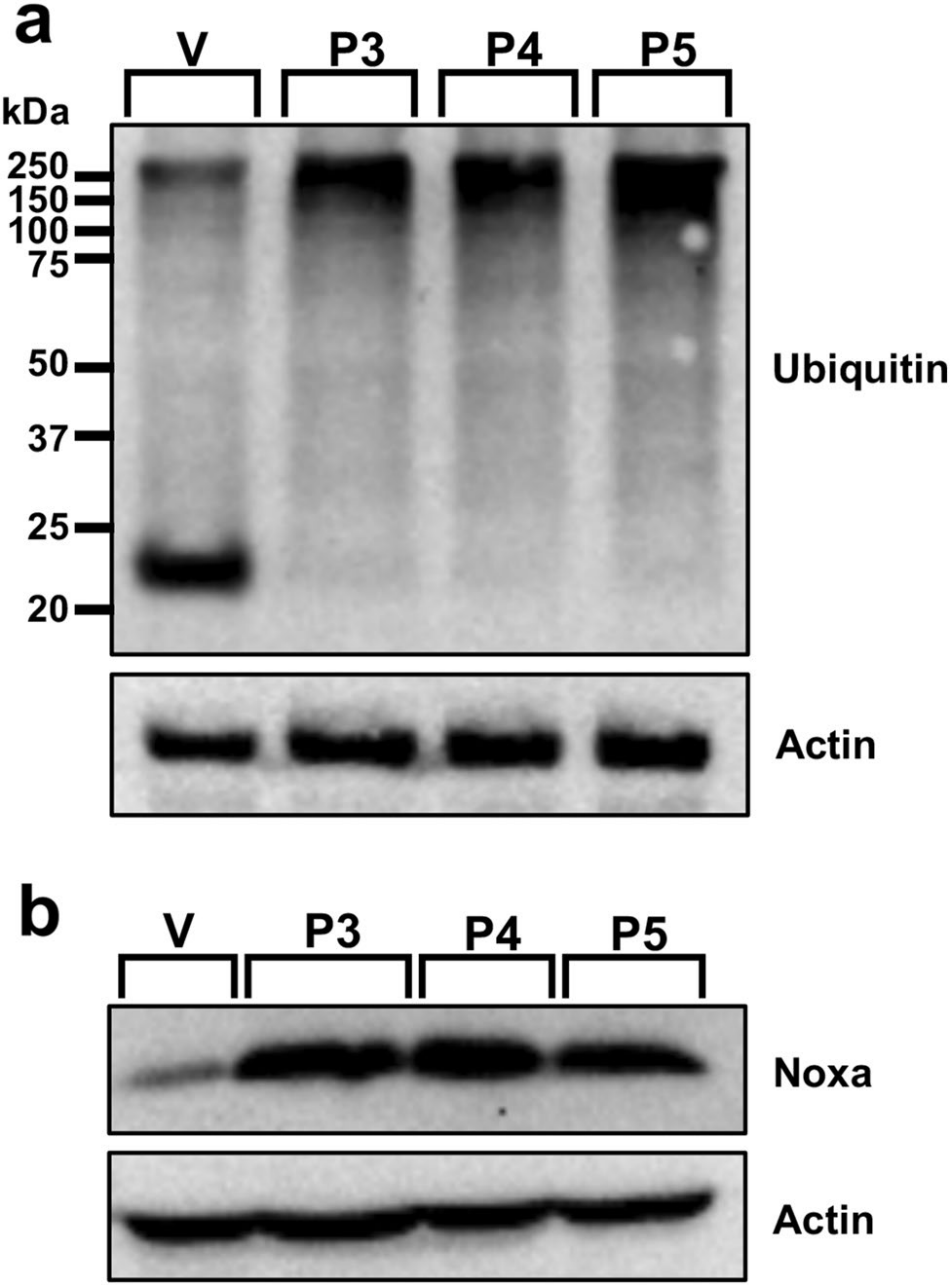
The accumulation of poly-ubiquitinated proteins and the pro-apoptotic protein Noxa is observed after treatment with P3–P5. Leukemic HL-60 cells were treated with vehicle (V; 0.3% v/v PEG-400), P3 at 3.34 µM, P4 at 4 µM, and P5 at 4 µM for 8 h. **a** An accumulation of high-molecular-weight poly-ubiquitinated proteins is observed after compound (lane 2–lane 4) treatment. **b** Increased Noxa protein expression is evident (lane 2–lane 4) following treatment with compounds. Actin was used as a housekeeping (protein loading) control. The identifiers for each lane are as follows: lane 1 = vehicle (V), lane 2 = P3, lane 3 = P4, and lane 4 = P5. Blots were cropped to focus on the area of interest. Original, unedited blots can be found in Supplementary File 2. The data presented are of one biological replicate (*n* = 1)

Next, we investigated proteasome inhibition by visualizing the accumulation of poly-ubiquitinated proteins [12]. Western blot analysis revealed the accumulation of high-molecular-weight poly-ubiquitinated proteins after treatment with P3 (0.54-fold), P4 (0.45-fold), and P5 (0.64-fold) when compared to vehicle control (Fig. 5a and Supplementary File 1). The pro-apoptotic protein Noxa is known to accumulate under conditions involving proteasome inhibition [32, 33]. P3 treatment induced a 2.54-fold increase, P4 treatment induced a 1.58-fold increase, and P5 treatment induced a 1.96-fold increase when compared to vehicle control (0.3% v*/*v PEG-400; Fig. 5b).

**Fig. 6:**
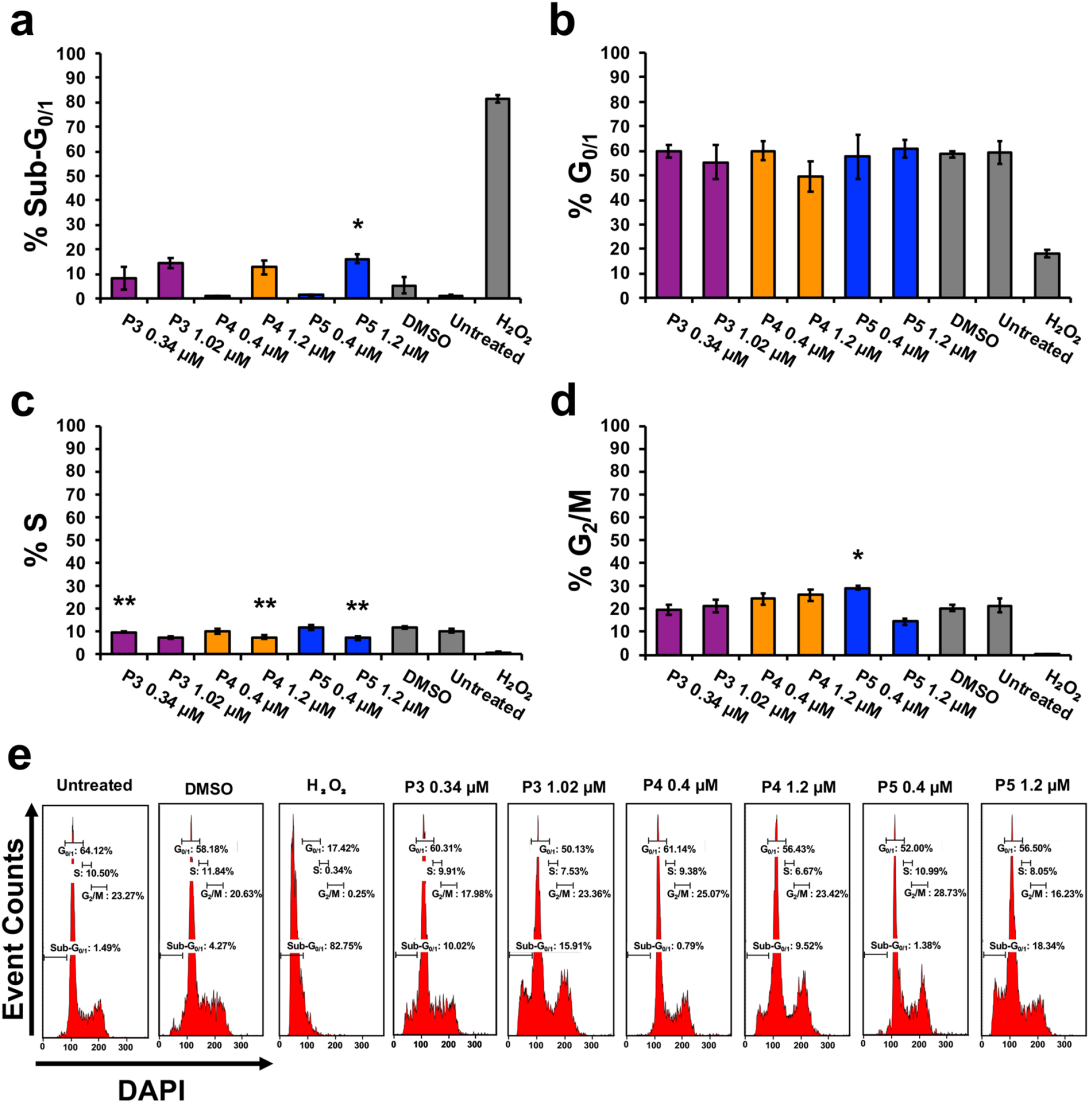
The cell cycle is disrupted with the treatment of compounds P3, P4, and P5. The phases of the cell cycle were visualized by measuring DNA content using NIM-DAPI and flow cytometry. **a**–**d** Graphs represent the percentage of HL-60 cells treated with P3, P4, and P5 for 72 h at the indicated concentrations. **a** Treatment with P3, P4, and P5 causes DNA fragmentation as observed by an increase in the cell population's percent in the Sub-G_0/1_ phase compared to vehicle control. **b** The G_0/1_ phase was not affected in a statistically significant manner after compound treatment. **c** There is a decrease of cells in the S phase after compound treatment. **d** Compounds P4 and P5 cause cell cycle arrest at the G_2_/M phase. **e** Representative histograms from flow cytometer analysis (approximately 10,000 events per sample) displaying the DNA content distribution after NIM-DAPI staining which corresponds to the different phases of the cell cycle. Data represent the mean ± SD (*n* = 3, except treatment P5 at 1.2 µM which is *n* = 2). Significant differences between the treatment groups and the vehicle control were assessed with the one-way ANOVA followed by the Bonferroni post hoc analysis (*p ≤ 0.05, **p ≤ 0.01)

Proteasome inhibitors are known to cause cell cycle arrest [34]. Therefore, we investigated the cell cycle profile after compound treatment. The Sub-G_0/1_ phase corresponds to cells with DNA fragmentation [23]. One-way ANOVA analysis revealed significant differences in the percentage of cells with DNA fragmentation between compound treatment groups and the DMSO control (Fig. 6a) (*F*_6,13_ = 11.36, *p* = 0.0002). Post hoc analysis revealed a significant increase of 11% of cells with DNA fragmentation after P5 treatment (0.4 µM). No significant differences were observed between treatment and control groups in the G_0/1_ phase (*F*_6,13_ = 2.74, *p* = 0.06). The S phase displayed significant differences between compound treatment and control groups (*F*_6,13_ = 18.08, *p* < 0.0001). Post hoc analysis revealed a significant decrease in the percentage of cells in the S phase after P3, P4, and P5 treatment at double the CC_50_ concentration as compared to DMSO (Fig. 6c). Significant differences were observed between the compound treatment and control group in the G_2_/M phase (*F*_6,13_ = 13.16, *p* < 0.0001). Post hoc analysis revealed a significant increase in the percentage of cells (~ 9%) arrested at the G_2_/M phase after P5 treatment (0.4 µM) when compared to the DMSO vehicle control (Fig. 6d). Increasing the dose of P5 (threefold) also led to a significant increase in cells arrested at the G_2_/M phase.

### P3–P5 causes the differential expression of genes important to their cytotoxic activity

**Fig. 7:**
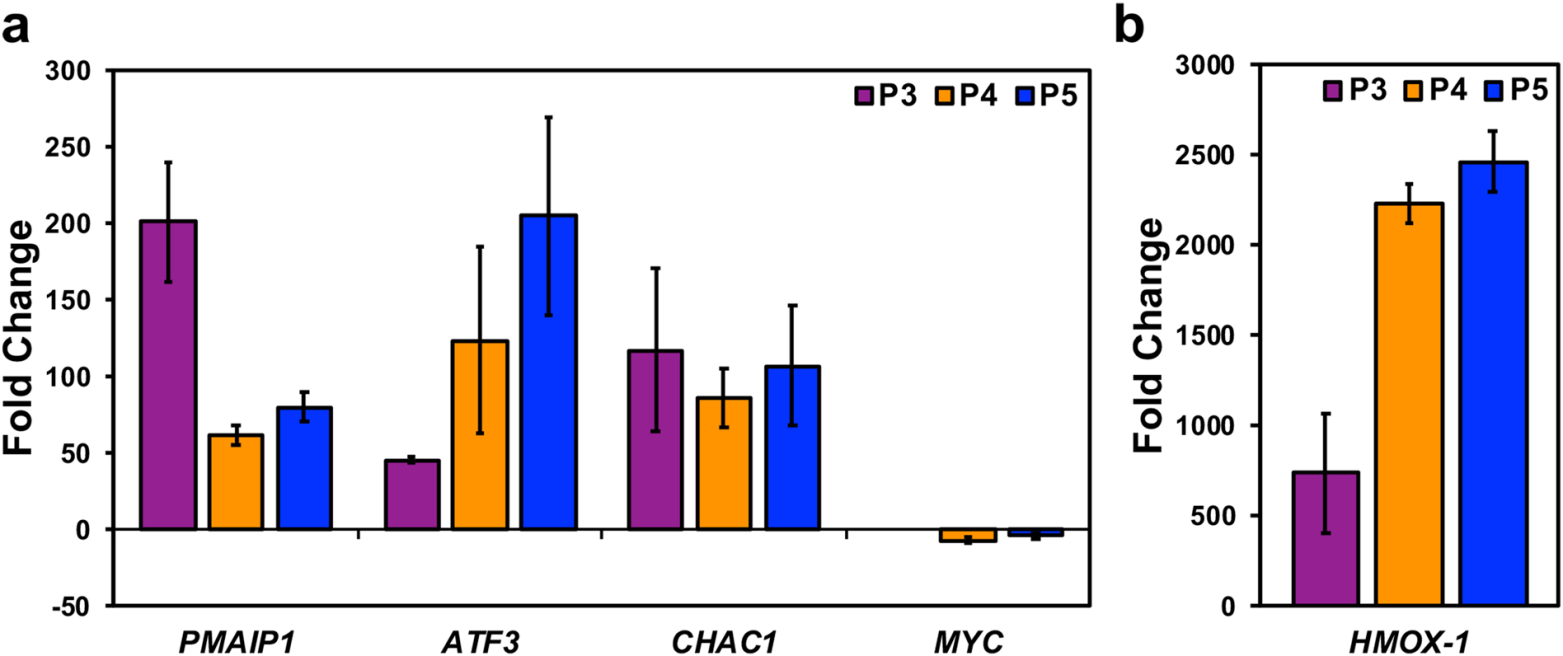
RT-qPCR data reveal differential regulation of genes important to the cytotoxic activity of P3–P5. *PMAIP1*, *ATF3*, *CHAC1*, *MYC*, and *HMOX-1* have roles in apoptosis, more specifically, stress-mediated cell death. **a**, **b** HL-60 cells were treated for 6 h with P3 (3.34 µM), P4 (4 µM), and P5 (4 µM). The fold-change differences of compound treatment relative to vehicle control (0.3% v/v PEG-400) are displayed on the *y*-axis. Each bar represents the mean fold change of three independent experiments (± SD). The comparative C_t_ method (2^−∆∆Ct^) was used to calculate gene expression and fold-change values. Gene expression levels were normalized to the housekeeping gene actin (*ACTB*). Data for *HMOX-1* are of two independent experiments

Finally, we examined the differential expression of *PMAIP1*, *ATF3*, *CHAC1*, *MYC*, and *HMOX-1,* which were discovered in the transcriptome analysis of the compounds (P1 and P2) that we previously characterized [8]. We sought to investigate the aforementioned genes because we believe they are related to processes relevant to the activity of these types of piperidones. These processes include the generation of stress that exploits the stress phenotype of cancer cells to induce apoptosis in malignant cells, and not normal cells [35]. The *PMAIP1* gene and its encoded protein Noxa are important apoptotic mediators [36]. *ATF3* is inducible after subjecting cells to oxidative and endoplasmic reticulum (ER) stress [37]. *CHAC1* plays a role in the unfolded protein response (UPR), which occurs as a response to ER stress and is also pro-apoptotic [38, 39]. *HMOX-1* is over-expressed after oxidative stress [40]. Overexpression of *MYC* helps cancer cells maintain their unregulated proliferative characteristics, and its down-regulation can reverse this effect [41]. We obtained the following fold-change differences after a comparative analysis of the normalized gene expression levels of compound-treated versus vehicle-treated samples. P3 treatment induced fold-change differences of 200.73 for *PMAIP1*, 45.32 for *ATF3*, 117.09 for *CHAC1*, and 737.96 for *HMOX-1* (Fig. 7a, b). *MYC* was not detected after P3 treatment. P4 treatment induced fold-change differences of 61.38 for *PMAIP1*, 123.50 for *ATF3*, 85.47 for *CHAC1*, -7.17 for *MYC*, and 2234.25 for *HMOX-1* (Fig. 7a, b). Finally, P5 treatment induced fold-change differences of 79.50 for *PMAIP1*, 204.56 for *ATF3*, 106.96 for *CHAC1*, -4.15 for *MYC*, and 2464.89 for *HMOX-1* (Fig. 7a, b).

## Discussion

In this study, we evaluated novel piperidone compounds P3, P4, and P5 (Fig. 1) to determine if they have similar cytotoxic properties as those previously published. The structures of P3–P5 differ only in the nature of the heterocyclic ring attached to the arylmethylene groups. These heterocycles are the pyrrolidine (P3), piperidine (P4), and morpholine (P5) groups. The pKa values of pyrrolidine, piperidine, and morpholine are 11.31, 11.12, and 8.50, respectively [42]. Hence, there will be more molecules of P5 existing as the free bases than is the case with P3 and P4, which may facilitate penetration of the cell membranes leading to greater toxicity. Such was the case, as P5 was the most toxic compound towards the nine cancerous cell lines (Table 1). Given the strong cytotoxic effects of these compounds on cancerous cells, we additionally examined the effects on non-malignant cells. Preferential cytotoxicity, denoted by SCI values above 1 (Table 1), towards cancer cells as opposed to non-cancerous cells was observed. Of the three compounds, P3 displayed the highest selectivity (SCI above 8) in most cell lines, except the colon COLO 205 and breast MDA-MB-231 cancer cell lines. Melphalan, a chemotherapeutic agent used in treating multiple myeloma (MM), leukemia, lymphoma, and other cancers, was tested in concert with the compounds of interest as a positive control [43]. Melphalan displayed significantly higher CC_50_ values than P3, P4, and P5 (Table 2), indicating that it has lower cytotoxicity than the piperidone compounds. Collectively, we believe P3–P5 are three potent compounds that are far more cytotoxic to cancer cells as opposed to non-cancer cells and with improved activity to that of a current chemotherapy drug.

Cytotoxic cancer therapies typically induce apoptosis [36, 44]. Thus, we decided to investigate if compounds P3–P5 induce apoptosis. A hallmark of apoptosis is the externalization of phosphatidylserine and the activation of the executioner caspase, caspase-3 [30]. Flow cytometry analysis revealed both phosphatidylserine externalization and caspase-3 activation after treatment with the piperidone compounds (Fig. 2). Although a significant increase of caspase-3 activation was not observed after P3 and P4 treatment, apoptosis can occur in a caspase-independent manner when the inner mitochondrial matrix is permeabilized [31]. Cells undergoing apoptosis display the morphological characteristic of DNA fragmentation [30]. P3–P5 caused DNA fragmentation of leukemic cells, identified as cells in the Sub-G_0/1_ phase (Fig. 6a). As previously mentioned, there are two main pathways of apoptosis: the intrinsic and extrinsic pathways [30]. The intrinsic pathway of apoptosis involves the mitochondria [30]. Mitochondrial membrane permeabilization causes a loss of the membrane potential (ΔΨm), which commits cells to apoptosis [31]. We observed mitochondrial membrane depolarization after treatment with compounds P3–P5 (Fig. 3). Additionally, ROS can play a role in initiating the intrinsic apoptosis cascade by being released after mitochondrial membrane depolarization [31, 45]. Treatment with the three compounds led to a large increase in ROS production (Fig. 4). Gene expression analyses also corroborate the induction of the intrinsic pathway of apoptosis. Compound treatment revealed the up-regulation of *PMAIP1* and *CHAC1*, as previously detected with compounds P1 and P2 (Fig. 7) [8]. *PMAIP1* is known as one of thirteen important genes for drug-induced intrinsic apoptosis [36]. *CHAC1* promotes apoptosis through the apoptosis-inducing factor-poly(ADP-ribose) polymerase (AIF-PARP) signaling cascade, which is essential in the intrinsic pathway of apoptosis [31, 39]. From these results, we can deduce that apoptosis, more specifically the intrinsic pathway of apoptosis, is the cell death mechanism induced by P3–P5 treatment.

Proteasome inhibition is an approach to exert tumor-specific therapeutic effects [46]. Bortezomib, the first clinically approved proteasome inhibitor, causes the accumulation of poly-ubiquitinated proteins, the accumulation of the pro-apoptotic protein Noxa, and gene expression alterations [47]. We previously discovered that piperidone compounds P1 and P2 induced these same cellular modulations; therefore, we investigated if the same was observed with P3–P5 treatment. As in our previous study, an accumulation of high-molecular-weight poly-ubiquitinated proteins with the treatment of P3–P5 was observed (Fig. 5a). Noxa is required for the activity of proteasome inhibitors in chronic lymphocytic leukemia (CLL), melanoma, and myeloma cells [33, 48, 49]. Cancer cells rapidly degrade Noxa due to its anti-proliferative characteristics, but proteasome inhibition can allow Noxa to accumulate [13]. The current evaluation revealed the accumulation of Noxa (~ 1.5, ~ 2, and ~ 2.5-fold differences), respectively, after P3–P5 treatment when compared to vehicle control (Fig. 5b). In addition to the components evaluated for P1 and P2, we examined the generation of ROS and cell cycle arrest after P3–P5 treatment to further investigate proteasome inhibition. ROS accumulate at early stages after treatment with proteasome inhibitors, as observed with Bortezomib in multiple myeloma cells [50]. Treatment with P3–P5 (Fig. 4) revealed a large accumulation of ROS. Proteasome inhibition can cause the accumulation of cell cycle regulators that cause cell cycle arrest to reverse certain inherent characteristics of cancer cells [34]. For example, proteasome inhibitors can revert the function of tumor suppressor p53 resulting in an arrest at the G_2_/M phase [34]. Bortezomib causes cell cycle arrest at the G_2_/M phase in non-small-cell lung cancer cells, colorectal cancer, and chronic myeloid leukemia (CML) [14, 34]. Another proteasome inhibitor, MG-132, causes G_2_/M cell cycle arrest in cervical and gastric cancer [34]. Analysis of the cell cycle profile after compound treatment revealed cell cycle arrest in the G_2_/M phase with the treatment of P5 (Fig. 6d). It is not clear why P3 and P4 did not cause cell cycle arrest at the G_2_/M phase, but it may be related to the lack or down-regulation of *c-Myc* expression (as shown in Fig. 7), which is an important gene for the proper function of the cell cycle [41]. Further analysis would have to be accomplished to understand this mechanism. Based on our data, we can deduce that our compounds behave as proteasome inhibitors.

Since we observed similar cytotoxic activity of compounds P3–P5 to that of P1 and P2, we decided to investigate if the same genes (*PMAIP1*, *ATF3*, *CHAC1*, *MYC*, and *HMOX-1*) were differentially regulated. Proteasome inhibitors can drive a cancerous cell to apoptosis by contributing to the endogenous stress phenotype [51]. The aforementioned genes are important to the development of proteotoxic stress, which can occur after proteasome inhibition, and the response of the cell to this stress [46, 49, 51, 52]. The same trend that was previously observed with compounds P1 and P2 of gene expression (up-and down-regulation) was also observed with the current piperidone compounds (Fig. 7). However, one difference was the lack of detectable expression of *MYC* after P3 treatment (Fig. 7). Repression of *MYC* results in the reversal of tumorigenesis in various in vivo mouse models, including T-cell acute lymphoblastic leukemia, hepatocellular carcinoma, osteosarcoma, among others [41]. Therefore, the down-regulation or complete loss of the *MYC* oncogene can reverse the uncontrolled replicative characteristic of cancer cells. In the case of compound P3, the lack of *MYC* expression can provide an additional therapeutic advantage that must be further evaluated. In addition to the importance of these proteins/genes in generating the stress to cause apoptosis, some modulate Noxa which we believe may be key to the activity of P3–P5. The Noxa promoter has *ATF3* and *MYC* binding sites, which can regulate its expression [53]. These gene expression analyses reinforce proteasome inhibition activity induced by our compounds.

We hypothesize that the molecular targets of the compounds of interest are deubiquitinating enzymes found within the 19S regulatory particle of the proteasome. Prior studies have shown that b-AP15, with a similar structure to piperidones P3–P5, shows inhibitory activity towards UCHL5 and USP14 and not towards other deubiquitinating enzymes found within the proteasome [54]. Through combined molecular docking and molecular mechanics, we evaluated the potential of our compounds to bind to the deubiquitinating enzymes UCHL5 and USP14. Although there is no consensus of threshold cutoff for docking studies, predicted binding energies are used to rank ligand–protein interactions [55, 56]. However, docking alone should not be used to predict the affinity of a ligand-substrate interaction [57]. Molecular mechanics is a follow-up analysis to molecular docking predictions [55, 58]. When used in combination, these are reliable predictive tools of ligand–protein interactions [58]. Successful use of these tools includes the scoring of various chemotherapeutic agents such as paclitaxel, gemcitabine, and cisplatin to investigate the protein MDR1 which contributes to multi-drug resistance [59]. In our studies, we performed molecular mechanics using Prime MM-GBSA from Schrodinger to calculate and rank the compounds based on binding affinity (kcal/mol) to improve the confidence of protein–ligand interactions observed in molecular docking experiments conducted with the Glide software. Since UCHL5 and USP14 could have many possible binding sites for these compounds, we explored various binding sites using sitemap generation included in Schrodinger. Sitemap ranks the likelihood of regions within a protein to be considered active binding sites. We took the best scoring sitemaps and performed docking and subsequent molecular mechanics simulations. Compound P2, which we evaluated in a previous study, was also included in these molecular docking and molecular mechanics studies since the current evaluation of P3–P5 was accomplished as a follow-up study [8]. The top scores (i.e., most negative) for UCHL5 (PDB: 4UEM) were for sitemap 2 (see Supplementary Fig. 2). The docking scores and binding affinities, respectively, to UCHL5 for P2 are -5.61 and -68.83 kcal/mol, for P3 are -5.12 and -65.44 kcal/mol, for P4 are -5.23 and-74.15 kcal/mol, and for P5 are -4.96 and -62.89 kcal/mol. Supplementary Fig. 2 displays the docking data of P2, P3, P4, and P5 bound to sitemap 2 of UCHL5. The top scores for USP14 (PDB: 6IIN) were for a known inhibitor site (see Supplementary Fig. 3). The docking scores and binding affinities, respectively, to USP14 are the following: P2 − 7.21 and − 45.64 kcal/mol, P3 -6.18 and -51.67 kcal/mol, P4 -5.86 and − 57.16 kcal/mol, and P5 − 5.16 and -58.45 kcal/mol. Supplementary Fig. 3 displays the docking data of P2, P3, P4, and P5 bound to the inhibitor site of UCHL5. From these data, we can predict that the piperidones being investigated bind to deubiquitinases UCHL5 and USP14. Based on docking scores, P2 displayed favorable results for having the strongest interaction with UCHL5 and USP14. However, the more reliable molecular mechanics analysis revealed that P4 and P5 had the best binding affinity to UCHL5 and USP14, respectively. In vitro data supporting inhibition of the proteasome, i.e., the accumulation of poly-ubiquitinated proteins (Fig. 6a), the up-regulation of Noxa (Fig. 6b), and the differential regulation of genes important to this activity (Fig. 7), supports the interaction of P4 with UCHL5. The in vitro results are also in concert with P5 binding USP14 to inhibit the proteasome since it displayed the highest fold-change increase (0.64) of poly-ubiquitinated proteins (Fig. 5a), up-regulation of Noxa (Fig. 5b), up- and down-regulation of important proteasome inhibition-related genes (Fig. 7), and finally cell cycle arrest at the G_2_/M phase (Fig. 6d). This was in addition to the strong apoptotic induction of P5 which had the lowest CC_50_ average against tumorigenic cell lines and a significant increase in the percentage of cells with phosphatidylserine externalization, caspase-3 activation, mitochondrial membrane depolarization, and DNA fragmentation. In the future, biochemical experiments need to be performed to conclude that the deubiquitinating enzymes UCHL5 and USP14 are the actual targets of these compounds.

## Conclusions

The behavior of compounds P3, P4, and P5 is in concert with the behavior of the previously published compounds P1 and P2 [8]. We observe apoptosis as the cell death mechanism activated by these compounds, specifically, the activation of the intrinsic pathway of apoptosis. Investigation of the mechanism by which these compounds induce apoptosis leads us to believe that the piperidone compounds can cause proteasome inhibition that leads to proteotoxic stress. There is a strong correlation between the genes and proteins affected by these compounds to that of proteasome inhibitors. These compounds appear to inhibit deubiquitinating enzymes within the proteasome as other similarly structured compounds do [16]. Given that clinically approved proteasome inhibitors can eventually become ineffective, these compounds could resolve resistance issues by targeting different components of the proteasome in combination with currently approved proteasome inhibitors [16, 60]. In conclusion, we have discovered that compounds P3, P4, and P5, display strong potential as anticancer agents to be explored in the future.

## Supplementary Information

Below is the link to the electronic supplementary material.

Supplementary file1 (DOCX 3965 kb)

Supplementary file2 (DOCX 14048 kb)

## Data Availability

All data generated or analyzed during this study are included in this published article [and its supplementary information files].

## Abbreviations

ANOVA: Analysis of variance
*ATF3*: Activating transcription factor 3
CC_50_: Cytotoxic concentration 50%
*CHAC1*: ChaC glutathione specific gamma-glutamylcyclotransferase 1
DMSO: Dimethyl sulfoxide
DNS: Differential nuclear staining
ER: Endoplasmic reticulum
FITC: Fluorescein isothiocyanate
H_2_O_2_: Hydrogen peroxide
*HMOX-1*: Heme Oxygenase 1
*MYC*: MYC proto-oncogene
NIM-DAPI: Nuclear isolation medium (NIM)- 4, 6-diamidino-2-phenylindole (DAPI)
PEG: Polyethylene glycol
PI: Propidium Iodide
*PMAIP1*: Phorbol-12-myristate-13-acetate-induced protein 1
PVDF: Polyvinylidene fluoride
ROS: Reactive oxygen species
RT-qPCR: Reverse transcriptase real-time polymerase chain reaction
SD: Standard deviation
TBS: Tris-buffered saline
UPR: Unfolded protein response
