# Supplementary material for: Three novel piperidones exhibit tumor-selective cytotoxicity on leukemia cells via protein degradation and stress-mediated mechanisms
Source: Pharmacol Rep. 2021 Aug 26;74(1):159–74. doi: 10.1007/s43440-021-00322-3 (PMC8786778; doi:10.1007/s43440-021-00322-3)
Supplement: Supplementary file 1 [file 43440_2021_322_MOESM1_ESM.docx]

**Pharmacological Reports**

**Three novel piperidones exhibit tumor-selective cytotoxicity on leukemia cells via protein degradation and stress-mediated mechanisms**

Lisett Contreras^1^, Stephanie Medina^1^, Austre Y Schiaffino Bustamante^1^, Edgar A Borrego^1^, Carlos A Valenzuela^1^, Umashankar Das^2^, Subhas S. Karki^3^, Jonathan R Dimmock^2^, and Renato J Aguilera^1^   

^1^Department of Biological Sciences and Border Biomedical Research Center, The University of Texas at El Paso, 500 West University Avenue, El Paso, TX, 79968-0519, USA.

^2^Drug Discovery and Development Research Group, College of Pharmacy and Nutrition, University of Saskatchewan, Saskatoon, S7N 5E5, Canada.

^3^Department of Pharmaceutical Chemistry, Dr. Prabhakar B. Kore Basic Science Research Center, Off-Campus, KLE College of Pharmacy, (A Constituent Unit of KAHER-Belagavi) Bengaluru-560010, Karnataka, India

Corresponding author e-mail: raguilera@utep.edu

**Table of Contents**

**Supplementary File 1**

1. **Supplementary Figure 1: Densitometry analysis of western blot images**

2. **Supplementary Table 1: Primer sequences for RT-qPCR**

3. **Supplementary Figure 2: 2D diagrams of compounds bound to UCHL5**

4. **Supplementary Figure 3: 2D diagrams of compounds bound to USP14**

**Supplementary Figure 1: Densitometry analysis of western blot images**. The Image Studio Lite (LI-COR) software was used for analysis. (a) Fold change values for ubiquitinated proteins above 75 kDa. (b) Fold change values for the Noxa protein. The following method was used to determine fold change values: the protein of interest was first compared to the protein loading control (actin), then, each compound (P3, P4, and P5) was compared to the vehicle control (0.3% *v/v* PEG-400). 

**Supplementary Table 1: Primer sequences for RT-qPCR.** Displayed are the forward and reverse primer sequences used in the RT-qPCR reactions. The final concentration of each primer (forward and reverse) in each PCR reaction was 700 nM. This information was previously published by our group (Contreras et al. 2018).

| **Primer** | **RefSeq** | **Forward 5`-3`** | **Reverse 5`-3`** | **Annealing Temp. for primer pair** |
| --- | --- | --- | --- | --- |
| **ATF3** | NM_001674 | TTG CAG AGC TAA GCA GTA GTG GTA | ATG GTT CTC TGC TGC TGG GAT TCT | 56°C |
| **CHAC1** | NM_024111 | CCT GAA GTA CCT GAA TGT GCG AGA | GCA GCA AGT ATT CAA GGT TGT GGC | 57°C |
| **MYC** | NM_002467 | CTT CTC TCC GTC CTC GGA TTC T | GAA GGT GAT CCA GAC TCT GAC CTT | 57°C |
| **HMOX1** | NM_002133 | CTC AAA CCT CCA AAA GCC | TCA AAA ACC ACC CCA ACC C | 50°C |
| **PMAIP1** | NM_021127 | GCT GGA AGT CGA GTG TGC TA | CCT GAG CAG AAG AGT TTG GA | 53°C |
| **ACTB** | NM_001101 | GGC ACC CAG CAC AAT GAA G | GCC GAT CCA CAC GGA GTA CT | 56°C |

 

**Citation:** L. Contreras, R.I. Calderon, A. Varela-Ramirez, H-Y. Zhang, Y. Quan, U. Das, J.R. Dimmock, R. Skouta, R.J. Aguilera, Induction of apoptosis via proteasome inhibition in leukemia/lymphoma cells by two potent piperidones. Cell. Oncol. (2018). doi: 10.1007/s13402-018-0397-1

**Supplementary Figure 2: 2D diagrams of compounds bound to UCHL5.** Shown are 2D diagrams of (a) P3 (b) P4 (c) P5 and (d) P2 interacting with the binding site (sitemap 2) of UCHL5 (PDB:4UEM). 

**Supplementary Figure 3: 2D diagrams of compounds bound to USP14.** Shown are 2D diagrams of (a) P3 (b) P4 (c) P5 and (d) P2 interacting with the inhibitor site of USP14 (PDB:6IIN).
