# Supplementary material for: Three novel piperidones exhibit tumor-selective cytotoxicity on leukemia cells via protein degradation and stress-mediated mechanisms
Source: Pharmacol Rep. 2021 Aug 26;74(1):159–74. doi: 10.1007/s43440-021-00322-3 (PMC8786778; doi:10.1007/s43440-021-00322-3)
Supplement: Supplementary file 2 [file 43440_2021_322_MOESM2_ESM.docx]

**Pharmacological Reports**

**Three novel piperidones exhibit tumor-selective cytotoxicity on leukemia cells via protein degradation and stress-mediated mechanisms**

Lisett Contreras^1^, Stephanie Medina^1^, Austre Y Schiaffino Bustamante^1^, Edgar A Borrego^1^, Carlos A Valenzuela^1^, Umashankar Das^2^, Subhas S. Karki^3^, Jonathan R Dimmock^2^, and Renato J Aguilera^1^   

^1^Department of Biological Sciences and Border Biomedical Research Center, The University of Texas at El Paso, 500 West University Avenue, El Paso, TX, 79968-0519, USA.

^2^Drug Discovery and Development Research Group, College of Pharmacy and Nutrition, University of Saskatchewan, Saskatoon, S7N 5E5, Canada.

^3^Department of Pharmaceutical Chemistry, Dr. Prabhakar B. Kore Basic Science Research Center, Off-Campus, KLE College of Pharmacy, (A Constituent Unit of KAHER-Belagavi) Bengaluru-560010, Karnataka, India

Corresponding author e-mail: [raguilera@utep.edu](mailto:raguilera@utep.edu)

**Supplementary File 2**

**Description:** Images of original, unedited blots obtained using the Thermo Fischer iBright 1500 instrument. The images were attained by exposing the polyvinylidene fluoride (PVDF) membranes for varying times that were calculated using the “Auto Exposure” function of the iBright instrument. Two separate gel electrophoresis experiments (Supplementary Figure 2 and Supplementary Figure 3) were conducted using the same protein samples obtained using the methods described in the manuscript. The second gel electrophoresis experiment (Supplementary Figure 3) was accomplished for visualization purposes and to remove samples that were not relevant to this analysis. 

**Supplementary Figure 4: Original, unedited image of the blot corresponding to Figure 5a.** (a) The blot was probed with anti-ubiquitin to detect high-molecular polyubiquitinated proteins. (b) The blot was probed with anti-actin to detect the loading control protein actin. The following edits were made to each blot (both a & b) to obtain the final images: crop of the last six lanes, horizontal flip, and slight rotation of the blot. The last six lanes were cropped because the samples used in this manuscript are in the first four lanes. Lane five corresponds to the molecular weight ladder that is shown by tick marks on the final image. The last five lanes (lane 6 to lane 10) correspond to samples from different treatments that are not relevant to the analysis accomplished in this manuscript. The other two adjustments were done for aesthetics. We did a horizontal flip so that the vehicle would be on the first lane and followed by the treatment (P3, P4, and P5). Once we did the horizontal flip, we slightly rotated the image to the left to straighten out the lanes.

**Supplementary Figure 5: Original, unedited image of the blot corresponding to Figure 5b.** (a) The blot was probed with anti-Noxa to detect the protein Noxa. (b) The blot was probed with anti-actin to detect the loading control protein actin. One modification was made to each blot (both a & b) to obtain the final images. There was a crop to only display the area where the protein of interest is located.
